# Infant colic, young children’s temperament and sleep in a population based longitudinal cohort study

**DOI:** 10.1186/s12887-022-03231-3

**Published:** 2022-03-30

**Authors:** Sølvi Helseth, Nina Misvær, Milada Småstuen, Randi Andenæs, Lisbeth Valla

**Affiliations:** Faculty of Health Sciences, Oslo Metropolitan University, Oslo, Norway

**Keywords:** Infant colic, Sleep-problems in children, Child temperament, Child health centres, Birth cohort, Longitudinal, MoBa

## Abstract

**Background:**

Colic and sleep problems are common among infants, constitute challenges and distress for parents, and are often reasons for seeking help from health professionals. The literature debates whether infant colic and sleep problems are linked together or not. Further, limited evidence exists on how colic impacts on child temperament and sleep during early childhood. Thus, the purpose of this study was to increase our knowledge of the characteristics of infants with a history of colic compared to infants without, and to study how infant colic is associated with the development of child temperament and sleep over time.

**Methods:**

The study is based on The Norwegian Mother, Father and Child Cohort Study (MoBa), a population-based cohort study conducted by the Norwegian Institute of Public Health. This sample contains 88,186 mothers and children. Data was retrieved from questionnaires distributed to mothers at recruitment (in pregnancy) and when the child was 6 months, 18 months, 3 years, and 5 years. Data was analysed using linear mixed models and GLM models for repeated measures.

**Results:**

At 6 months, infants with reported colic are described as fussier, present more sleeping problems, are breastfed less, and the families visit the child health centre more often when compared to the non-colic group. Mothers of children with reported colic perceive their children’s temperament significantly more challenging from the age of 6 months to 5 years. Further, children with reported colic were more likely to sleep less than recommended (22%) and to have more frequent night awakenings (14%) than usual for their age (6 months to 5 years).

**Conclusion:**

Infant colic often occurs together with other signs of regulatory problems which may amplify the load on the parents. Moderate differences in temperament and sleep-problems across time, between those with colic and those without, indicate that the diagnosis of colic is moderately associated with later behavioural difficulties. However, it is demanding for the parents, and important to be aware of and act upon symptoms of colic in the child health centres to reduce the parents’ load and prevent adverse long-term outcomes**.**

## Background

Problematic infant fussing and crying, often called infant colic, is a well-known condition among parents, causing them stress and concern. Despite its commonness, the cause of colic is still debated, effective treatment is lacking and the criteria for defining it are vague. The occurrence varies in different studies depending on the criteria used, however a systematic review has shown a mean prevalence between 17 and 25% across the world [1]. Infant colic usually occurs in the infant’s second or third week of life [2], persists at a high level (117–133 min a day) during the first 6 weeks, and then gradually resolves in the third or fourth month [1]. The crying is often described as persistent and more than is normally expected [3]. It might be questioned what a normal amount of crying and fussing is, and mostly it is the parents’ experiences and tolerance that determines when the crying is perceived to exceed normal [3]. However, when crying and fussing exceeds 2–3 h a day, it will usually be characterized as more than normal, both by parents and health professionals [2]. The most common definition of infant colic is Wessel’s “rule of threes”: “the infant fusses or cries more than three hours a day, more than three days a week, and for more than three weeks, but is otherwise healthy” [4]. The “rule of threes” does not point to any specific cause of colic. Although there are many theories, there is currently no consensus on the cause in the literature. The most frequently described causes are gastrointestinal disorders, infant characteristics (personality), immaturity of the central nervous system, parent-child interaction, and an extreme degree of normal distress [5].

Infants’ sleep/wake patterns will settle during the first year of life. According to recommendations, 12–16 h per 24 h is considered to be a normal amount of sleep for infants from 4 months to 12 months [6, 7]. At 4 months, 60–70% of infants sleep continuously for at least 5 h during a regular night [8, 9]. Henderson, France [8] found that this also applied for most 2–3 months old infants. The majority of infants wake up during the night; however, problems first occur when infants signal parents upon wakening rather than resettling themselves [10]. Younger infants more often require parental intervention to return to sleep, than older. However, when 12 months old, some infants still need parents to intervene to get back to sleep [10]. St James-Roberts and Peachey [11] claim that, in most cases, sleeping problems in infancy involve a delay in ending night-time signalling behaviour (crying) and can sometimes be explained by looking at the parenting environment. Night-waking and signalling, in many cases, can be prevented with a limit-setting parenting style [5]. While infant colic is most prominent during the infant’s first 6 weeks, infant sleeping problems appear later in infancy [11].

The literature is not consistent about whether infants who cry a lot in the first weeks of life also tend to have sleeping problems. St James-Roberts [3] claims that, in most cases, infant crying and sleeping problems are distinct cases. However, there is evidence that for some infants, fussing and crying (colic), sleeping problems, and feeding difficulties occur together, and are described in the literature as infant regulatory problems [12, 13]. The concurrent appearance of such problems increases the load on the parents and is associated with future mental health concerns of the child [14], and is thus important to study further.

The research on long-term consequences that infant crying has for the child is sparse. Some evidence exist that shows excessive crying or infant colic increases the risk of mood and behavioural problems in pre-school and early school age [15–17], while the associations between infant colic and children’s sleep across the first 5 years of life, as far as we know, remains to be studied. As knowledge on how infant colic is associated with temperament and sleep later in childhood is limited, these relationships warrant further studies. Health care providers would benefit from a greater understanding of how colic can impact the infants’ future development, which in turn should have consequences for the follow-up of young families in child health centres.

The overall aim of this study was to increase our knowledge of the characteristics of infants with a history of colic and to study how colic in infancy is associated with the development of child temperament and sleep behaviour over time. Such knowledge will enable early, targeted interventions to support parents and children and prevent possible negative consequences of colic. More specifically, the research questions are:

- To compare characteristics of infants (6 months) with a history of colic with infants without a history of colic on selected variables such as child gender, child temperament, child sleep, pregnancy duration, breastfeeding, visits at the child health centre, mothers’ experience of birth and mothers’ education
- To describe the associations between infant colic and the development of young children’s temperament over time (from when the child is 6 months until 5 years), and
- To estimate how infant colic is associated with young children’s sleep (duration and quality) over time.

## Methods

### Study population

The Norwegian Mother, Father and Child Cohort Study (MoBa) is a population-based pregnancy cohort study conducted by the Norwegian Institute of Public Health. The MoBa is a study of health and causes of disease among mothers and children. Participants were recruited from all over Norway from 1999 to 2008. All pregnant women in Norway were eligible for participation in MoBa if they were able to read Norwegian [18], and 41% of the pregnant women consented to participation. The cohort includes approximately 114.500 children, 95.200 mothers and 75.200 fathers. The current study is based on version 10 of the quality-assured data files released for research in 2017. The establishment of MoBa and initial data collection was based on a license from the Norwegian Data Protection Agency and approval from The Regional Committees for Medical and Health Research Ethics. The MoBa cohort is currently regulated by the Norwegian Health Registry Act. The current study was approved by The Regional Committees for Medical and Health Research Ethics (2017/2199/REK-SØ) and the Norwegian Centre for Research Data (NSD 309313).

For the current study we have analysed data of 88,186 mothers and children. Twins (*n* = 1480) and triplets (*n* = 14) were excluded.

### Data collection

Biological material from mothers, and children and questionnaire data, have been collected since pregnancy and until the child is 8 years old, making the MoBa-study unique [18]. The questionnaires ask about health and illness in mothers and their children at given time points. The mothers complete the questionnaires during pregnancy (16–20 weeks), and when the child is 6 months and 18 months, and 3, 5, and 8 years [18]. For the purpose of the present study, we used data from Questionnaire 1 (Q1 at recruitment), Questionnaire 4 (Q4, Child is 6 months), Questionnaire 5 (Q5, Child is 18 months), Questionnaire 6 (Q6, Child is 3 years), and Questionnaire 7 (Q7, Child is 5 years).

### Measures

Data on child gender, birth weight and gestational age, mother’s age, mother’s educational level, civil status and work status, breastfeeding, visits at child health centres, and mother’s experience with birth were collected from the questionnaires Q1 (16–20 weeks of pregnancy) and Q4 (6 months postpartum).

Infant colic is measured with one question in Q4 (at 6 months): - Has your child had the following illness/health problem? Infant colic? Yes or No. Colic is listed as number 11 out of 12 common health problems during infancy, such as cold, ear infection, and false croup. No description or explanation about colic supplemented the question. Thus, whether the child has had colic or not is reported and interpreted by the mothers answering the questionnaires.

Sleep is assessed with questions concerning the amount of sleep (“How many hours in total does your child sleep per 24-h period?”), night awakenings (“How often does your child usually wake during the night?”), and a question about how easy the child is to put to bed at night (“The child is easy to put to bed and falls asleep quickly”)*.* Questions on sleep at 6 months (Q4), 18 months (Q5), and 5 years (Q7) are used in this study. Sleep was not included in the questionnaire when the child was 3 years old (Q6). The response options for sleep duration were; less than 8 h, 8–10 h, 11–12 h, 13–14 h and more than 14 h, and for night awakenings; 3 or more times every night, once or twice every night, a few times a week and seldom or rarely The variables on quantity and night awakenings were dichotomized into normal and not normal based on recommendations in the literature [6, 7]. We asserted that a normal amount of sleep (or sleep as recommended) of a child is 13 h or more a day at 6 months, 11 h or more a day at 18 months, and 10 h or more at 5 years [6, 7]. Based on Hysing et al. [6] we defined usual number of night awakenings as two times or less at 6 months, as 2 times or less at 18 months, and as sometimes or rarely at 5 years. The final sleep item “easy to put to bed and falls asleep quickly” was dichotomized into *easy* to put to bed and *not easy* to put to bed (only in Q4).

Child temperament was measured at 6 months (T0), 18 months (T1), 3 years (T2), and 5 years (T3). At T0 the Infant Characteristics Questionnaire (ICQ6) was used and at T1, T2, and T3 The Emotionality, Activity, and Shyness questionnaire (EAS) was used [19, 20].

The ICQ6 comprises 24 items on infant behaviour which constitute four subscales—Fussy/Difficult, Unadaptable, Dull, and Unpredictable—with varying internal consistency reported (.79, .75, .39, .50, respectively) (Bates et al., 1979). In MoBa only the Fussy/Difficult scale with eight items is used as a measure of temperament when the child is 6 months old. Mothers rank the item on a 7-point Likert scale [1–7], representing the level of perceived difficulty. Negatively framed questions were reversed, so that lower scores mean that the child is perceived as more fussy/difficult. The items were combined into a mean score and the internal consistency was 0.69.

The EAS questionnaire is recommended for children 1–9 years and consists of 20 items. Four subscales measure different temperament dimensions, Emotionality (irritability/anger), Activity (activity level), Sociability (positive affect/including approach), and Shyness (fear)—and are described with five items each. The mothers are asked to rate whether the different statements apply to their child using five response categories from “very typical to “not at all typical” [1–5]. Positively framed questions were reversed so that lower scores mean the child’s temperament is perceived more challenging. In the Norwegian MoBa study, a short form of EAS with three items in each scale was used. A mean index is calculated from the items of each scale. The reliability and validity of the EAS have earlier been established with data from the MoBa study with satisfactory results [20]. In our study the averaged estimates of Cronbach’s alpha were 0.73 for Emotionality, 0.64 for Activity, 0.60 for Sociability and 0.75 for Shyness, meaning moderate to good reliability.

### Data analyses

Continuous variables are described with mean and standard deviation (SD) and categorical variables with counts and percentages. Crude differences between groups are analysed using t-test (for continuous data) or chi-square test (for categorical data). Possible differences in infant temperament between groups (infants with and without colic) over time and at given time points were analysed using linear mixed models for repeated measures with an unstructured covariance matrix to account for statistical dependencies as the same children were assessed several times. Linear mixed models use all available data and do not require imputation of missing values, thereby limiting any possible bias caused by dropouts. All models were adjusted for maternal education, gestational age, and mother’s age. The results are expressed as estimated means for each time point with 95% confidence intervals (CI). In addition, the estimated differences between the means of infants with and without a history of colic at 6 months were calculated for each time point.

To estimate how colic is associated with the amount of sleep and frequency of night awakening, we have fitted Generalized Linear Models (GLM) for repeated measures with a logit link. Both outcomes were dichotomized, e.g., number of hours slept, and frequency of awakening as considered normal for a given age. In addition to modelling having had a history of colic or not at 6 months, all models were adjusted for child gender, gestational age, maternal education, and mother’s age. The results are expressed as odds ratio (OR) with 95% confidence intervals (CI).

All tests were two sided and *p*-values < 0.01 were considered statistically significant. The level of significance was lowered to 0.01 to account for multiple testing. Given the large sample size, there was a high risk of type I error so we focused on the point estimates and CIs instead of presenting *p*-values. All analyses were conducted using SPSS version 26.

## Results

### Characteristics of infants (6 months) with a history of colic compared with infants without

The prevalence of infants with a history of colic in this study is 11.9%. Table 1 describes the differences in the study population on various variables between infants with a history of colic and their mothers and those without Significant differences between the groups are found on all variables. Boys and infants born pre-term are more frequently in the colic group. At 6 months of age, infants with a history of colic are described as fussier, they sleep on average less and with more night awakenings, and they are more difficult to put to bed compared to infants without a history of colic. Infants that are exclusively breastfed the first 3 months of life (during the colic period) are rarer in the colic group. The differences persist until the infants are 6 months as the proportion of children who are still breastfed daily is smaller in the group who have had colic compared to those who had not. It is also notable that mothers in the colic group more often report a negative response to the question “I felt safe and in good hands” during birth. Lastly, mothers with infants that have a history of colic visit the child health centre more frequently and the proportion of those with more than 10 visits was significantly higher for mothers with infants with a history of colic (10.1%) than those without (6.9%).

**Table 1:** Some characteristics of infants with (11.6%) and without (88.4%) a history of colic and their mothers when the child is 6 months, including gender, gestation age, birthweight, patterns of sleep, fussiness (temperament), the mothers´ age, education, and experience at birth. (*N* = 88,186; missing = 3433; analysed = 84,753)

	Colic *n* = 9794 11.6%		Not colic *n* = 74,959 88.4%		
	Mean	SD	Mean	SD	***P***-value
Birth weight (gr)	3507.5	586.1	3593.8	571.0	**< 0.001**
Birth length (cm)	50.0	2.54	50.4	2.47	**< 0.001**
Mother’s age	29.6	4.7	29.8	4.5	**< 0.001**
ICQ - temperament/fussiness	3.69	0.48	3.87	0.44	**< 0.001**
	**n**	**%**	**n**	**%**	
Gender					**< 0.001**
• Boy	4718	52.9	34,915	50.8	
• Girl	4196	47.1	33,866	49.2	
• missing	880	9.0	6178	8.3	
Mothers’ education ^a^					**< 0.001**
• low	3329	34.0	23,717	31.6	
• medium	3758	38.4	29,785	39.7	
• high	2090	21.3	17,069	22.8	
• missing	617	6.3	4388	5.9	
Pregnancy duration					**< 0.001**
• normal ≥37 weeks	7971	92.6	62,811	94.6	
• born too early	640	7.4	3599	5.4	
• missing	1182	12.1	8549	11.4	
Safe delivery – mothers’ experience ^b^					**< 0.001**
• positive experience	7615	80.3	62,248	85.2	
• negative	1871	19.7	10,845	14.8	
• missing	308	3.1	1866	2.5	
Breastfeeding now					**< 0.001**
• sometimes or never	2240	23.3	14,340	19.4	
• daily	7378	76.7	59,587	80.6	
• missing	176	1.8	1032	1.4	
Exclusive breastfeeding (0–3 months)					**< 0.001**
• yes	5407	55.2	46,359	61.8	
• no	4387	44.8	28,600	38.2	
Child Health Centre (number of visits)					**< 0.001**
• 3–5 visits	3544	40.2	31,370	46.2	
• 6–10 visits	4275	48.5	31,309	46.2	
• more than 10 visits	987	10.1	5152	6.9	
• missing	988	10.1	7128	9.5	
Total hours of sleep at 6 months					**< 0.001**
• less than 13 h	517	5.4	2199	3.0	
• 13 h or more	9067	94.6	71,246	97.0	
• missing	210	2.1	1514	2.0	
Easy to put to bed					**< 0.001**
• easy	5925	71.3	55,588	85.1	
• not easy	2381	28.7	9728	14.9	
• missing	1488	15.2	9643	12.9	
Night awakenings					**< 0.001**
• usual (2 times or less)	2147	24.2	20,974	30.6	
• more than usual (3 times or more)	6723	75.8	47,606	69.4	
• missing	924	9.4	6379	8.5	

^a^ Education at 16–20 weeks of pregnancy: low education = completed elementary school and or high school. Medium education = completed college/university 1–3 years. High education = completed college/university ≥4 years

^b^ Experience of birth – "I felt safe and in good hands"

### The associations between colic and infant temperament over time

Multiple linear mixed models with repeated measures were fitted with infant colic as the independent variable and infant temperament as the dependent variable. The model was further adjusted for mothers’ age, education (in three categories), assessment time, pregnancy duration (pre-term versus full term), child’s gender, having colic (yes vs no) and the child’s sleep behavior (as recommended or not). We found that mothers with the lowest education scored significantly lower on infant temperament (B = -0.03; 95%CI [− 0.04; − 0.02]) compared to mothers with a medium or high level of education. There was no statistically significant difference in outcome between mothers with medium and high education. Mothers with children born pre-term scored significantly lower on infant temperament (B = -0.03; 95%CI [− 0.05; − 0.02]) compared to those who had children born full term. Maternal age was statistically significantly associated with higher scores on the outcome (B = 0.01; 95% CI [0.01; 0.01]) indicating that older mothers tend to rate their children as less temperamental.

**Fig. 1:**
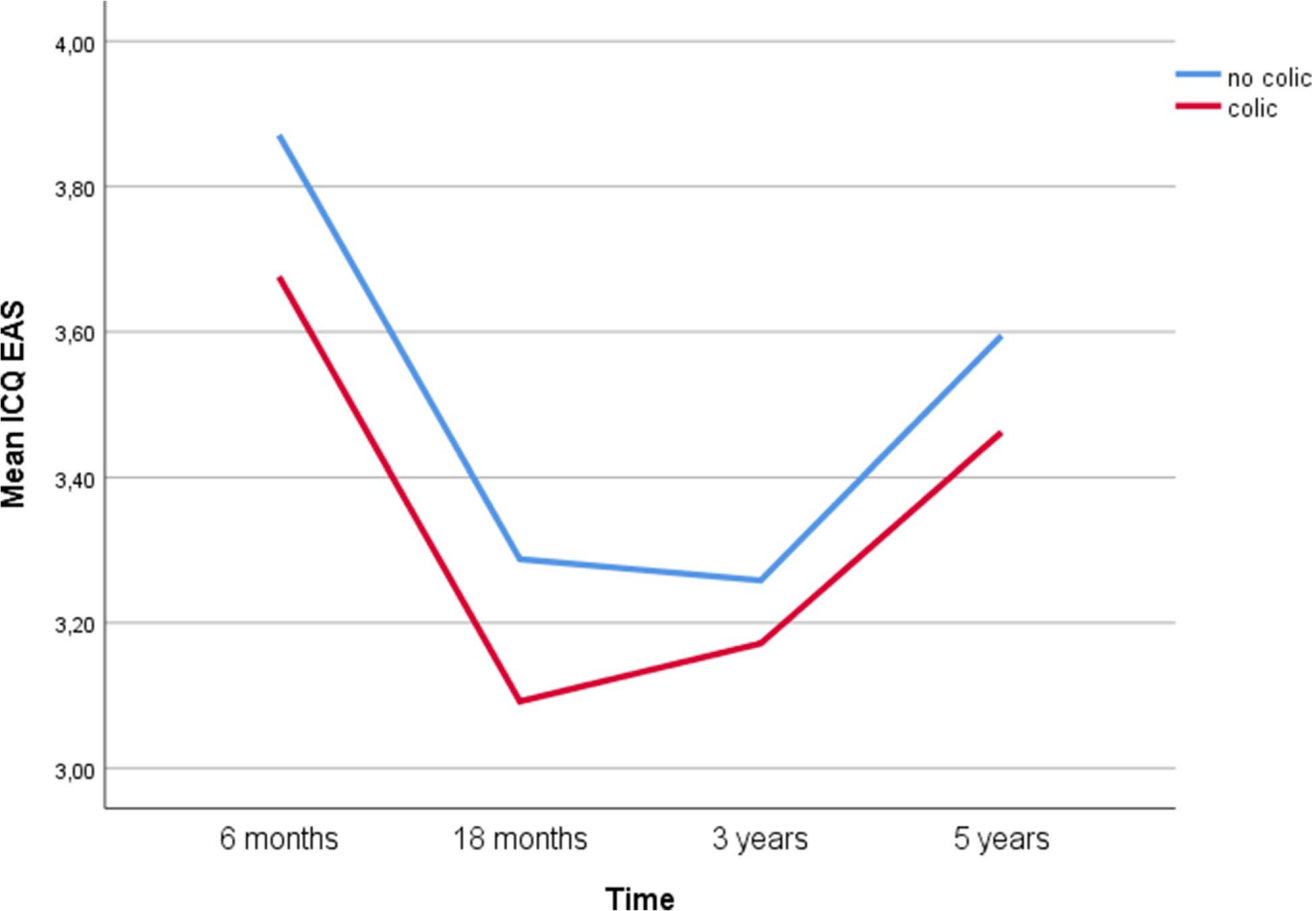
Graphical presentation with observed means of the associations between infant colic and child temperament over time

Table 2 and Fig. 1 show that infant colic has a lasting, but small, effect on child temperament over time. Children with reported colic had a significantly lower score on the temperament scale than those without at all points of measurement, meaning that the mothers perceive the children’s temperament more challenging from the age of 6 months to 5 years. The differences between the two groups persist, but as shown in Fig. 1, the difference is largest for the youngest children (6 months and 18 months) and decreasing from there.

**Table 2:** The associations between infant colic and child temperament over time (statistically significant differences are highlighted in bold)

Time	History of infant colic	Estimated mean	95% CI	Estimated between groups differences
6 months	No Colic	3.86	3.86; 3.87	**0.19**
	Colic	3.67	3.66; 3.68	
18 months	No Colic	3.27	3.27; 3.28	**0.19**
	Colic	3.08	3.06; 3.10	
3 years	No Colic	3.24	3.24; 3.25	**0.08**
	Colic	3.16	3.15; 3.18	
5 years	No Colic	3.58	3.57; 3.59	**0.12**
	Colic	3.46	3.44; 3.49	

- Adjusted for mothers’ age, education, pregnancy duration and child’s gender

- Dependent: ICQ_EAS

### The likelihood of having sleeping problems across time when the child has a history of colic

We have estimated the associations between having had colic and total amount of sleep and frequency of night awakenings (Table 3). Both outcomes were dichotomized, e.g., number of hours slept, and rated night awakenings as considered normal for a given age. Both multivariate models were adjusted for child gender, gestational age, maternal education, and mother’s age. The children with reported colic were significantly more likely (22%) to sleep less than recommended for their age (6 months to 5 years) and significantly more likely (14%) to have more frequent night awakenings than usual for their age (6 months to 5 years). Children with colic had lower odds of sleeping for the recommended number of hours compared to children without colic, reflecting the whole follow-up period (OR = 0.78, 95% CI [0.74; 0.81]). The differences between boys and girls in number of hours slept were statistically significant, however not clinically relevant (OR = 1.04, 95%CI [1.01; 1.07]). Further, the children with colic had higher odds of more frequent night awakenings than usual for their age during the whole follow-up period (OR = 1.14, 95% CI [1.09; 1.20]). In addition, girls were about 11% less likely to have frequent night awakenings (as considered normal for their age) compared to boys (OR = 0.89, 95%CI [0.86; 0.91]).

**Table 3:** The odds of having sleeping problems across time when the child has a history of colic, presented as odds ratio (OR) with 95% Confidence Intervals (CI) (significance highlighted in bold)

Variable	Frequent night awakenings			Number of hours slept		
	OR	95%CI	*P*-value	OR	95%CI	*P*-value
Colic (ref = no)	1.14	1.09; 1.20	**< 0.001**	0.78	0.74; 0.81	**< 0.001**
Gestational age	1.03	1.03; 1.04	**< 0.001**	0.99	0.99; 1.00	**0.007**
Gender (ref = boys)	0.89	0.86; 0.91	**< 0.001**	1.04	1.01; 1.07	**0.007**
Mother’s age	1.00	1.00; 1.01	0.064	0.97	0.97; 0.98	**< 0.001**
Mother’s education	1.20	1.18; 1.21	**< 0.001**	1.13	1.12; 1.14	**< 0.001**
Time	3.57	3.48; 3.67	**< 0.001**	0.54	0.52; 0.54	**< 0.001**

## Discussion

In this study, the prevalence of infant colic is 11.9%, lower than reported in a recent systematic review showing a mean prevalence between 17 and 25% [1]. Differences in reported prevalence of infant colic is often explained by ways of defining colic and how it is assessed, and whether parent-reported, diagnosed by health professionals or measured with valid instruments. In a systematic review, only four tools for colic assessment were obtained; they all had limitations and the need for developing new and valid scales was highlighted [21]. Thus, most studies rely on parent-reported data on colic which includes many sources of error. In MoBa, colic is assessed through one single recall question when the child is 6 months old, and the colic period is passed. It relies on the mothers’ perception of their infant’s behaviour, how they remember the past period, their knowledge on infant colic and whether they have received the diagnoses from a health professional. Mothers may over- or under-report colic, but their perceptions have been found to be reliable [22]. The fact that data on colic were collected after the colic period might partly explain why the prevalence is lower than expected. On the other hand, the recall period was short and a Swedish study showed that the memories of colic are vivid even 4 years after the colic period [23]. Thus, it is relevant to assume that parents will label it as colic when they define crying as a problem in the child’s first weeks. The parents’ subjective perceptions of what colic is relate to how colic is described in the literature with unsoothable bouts of crying and fussing interpreted as a sign of infant distress, which again causes parental frustration and stress [24]. The MoBa study does not contribute any criteria or explanation of colic to guide parents in reporting the condition but has listed the question in a series of questions on common illnesses during infancy. If parents did interpret their infants’ crying as colic or did not consider colic an illness, they may not have reported it, thereby explaining the rather low prevalence of colic in the study.

In this study, there are significant differences between the colic group and the non-colic group on all variables. At 6 months of age, infants with a history of colic are rated as fussier and more challenging (temperament), they present more sleeping problems, they are breastfed less, and the families visit the child health centre more often than the non-colic group. Given the big sample size in this study, small differences might be significant and thus, the results should be interpreted with caution. However, the results imply that, for some infants, fussing and crying (colic), sleeping problems, and possible feeding difficulties (lower incidence of mothers breast-feeding in the colic group) occur together, a triad described in the literature as infant regulatory problems [12, 13]. In a study by Cook, Mensah [12], more than 53% of the parents reported one regulatory problem at 1 month of age and 7.3% of the parents reported the whole triad of regulatory problems. For mothers reporting two problems occurring together, sleeping and crying-and-fussing were most frequently described (11.7%) [12]. A longitudinal study on infant regulation and child mental health concerns found that infants with severe regulatory problems have much greater odds of experiencing mental health concerns at 5 and 11 years [14].

The Norwegian MoBa study asserts that, when infant colic is reported, mothers also more frequently describe the appearance of other regulatory issues, like feeding difficulties and problems with sleep. However, we have not studied the prevalence of the problems occurring together or the severity of regulation problems. Instead, we have studied how having experienced one of these regulatory problems (infant colic) is related to child temperament and sleep over time. As a result, we see that the temperament of children with a history of infant colic is perceived as more challenging by their mothers from the age of 6 months until 5 years than those without colic. The biggest difference between the two groups, and maybe the most challenging period, appears when the child is 18 months. Several studies support the finding that infants with colic remain more temperamental during childhood [15–17]. Studying the impact of colic on sleep behaviour from 6 months to 5 years, we found that the children with reported colic were more likely to sleep less than recommended and to have more frequent night awakenings. The odds for sleeping less than recommended were higher than the odds for more frequent night awakenings. However, other studies have shown that frequent night awakenings are correlated with parental perceptions of a sleep problem while sleep duration is not [25].

The literature debates whether infant colic and sleep problems are linked together or not [11], but there is growing evidence that infant regulatory problems can occur as a single problem or as multiple regulatory problems [26]. When they occur together in early infancy, the odds that they persist when the child is nearly a year old is found to be threefold [27]. In addition, multiple regulatory problems are found to predict dysregulated behaviour across childhood [28]. In our study, we have shown an association between infant colic and other regulatory problems, such as problems with sleep, at 6 months. Further, we have shown that children with a history of colic are perceived as having a more challenging temperament by their mothers and have higher odds of being perceived as having sleep problems across time. However, the differences between those with and without colic appear to be moderate to small over time (6 months to 5 years). In many cases, regulatory problems—especially if there is only one—are transient and there is evidence that if colic resolves as expected when the infants are 3–4 months old, then the adverse effects on child temperament and behaviour are limited [29]. Still, the evidence that multiple regulatory problems often appear together and persist over time underlines the need for awareness of symptoms and close follow-up of the families. Infant crying and sleeping problems are a frequent cause of help-seeking from parents. In our study, we have shown that mothers who report colic visit the child health centres significantly more frequently than those that do not report colic. Since parenting is regarded as having an influence on the infants’ regulatory problems [12, 27], interventions should target parents and support and educate them in handling infants’ regulatory adaptation before the problems settle. There is evidence that interventions targeting overall parenting are effective in helping children with regulatory problems and their parents [5, 12]. Reported effects are reduced parental frustration anxiety, reported amount of infant crying, more contact with health professionals and increased knowledge about crying [30]. As described, the differences between the groups in this study are significant, but also moderate to small over time. The clinical significance should be more thoroughly investigated in future studies, however, our findings support previous research that infants with colic or excessive crying, alone or together with other regulatory problems in early infancy, have an elevated risk of later regulatory problems [26–28].

Whether infant colic should be termed a regulatory problem raises an important discussion. Is it colic itself or the most prominent symptom of colic, excessive crying and fussing, that could be labelled a regulatory problem? If colic is a regulatory problem, this points to an understanding of colic as a behavioural condition and extreme variant of normal crying which could be explained by some of the existing theories of infant colic, like infant personality, immaturity of the CNS, parent-child interaction, or a high degree of normal distress [5]. A well-known researcher on infant colic claims, “Colic may be best viewed as a clinical manifestation of normal emotional development, in which an infant has diminished capacity to regulate crying duration” [31]. However, this leaves out the theory of colic being a gastrointestinal disorder. The gastrointestinal causes of colic are often referred to as developmental lactose intolerance, altered gut microorganism, immaturity of the gut, increased motilin receptors, or cow milk hypersensitivity [32]. Yet, research has shown that gastrointestinal disturbances only apply to a minority (5%) of infants who cry excessively during the first months of life [5, 30]. Still, it is important to be aware that the term colic is used as a collective term for excessive infant crying and fussing. Thus, a thorough assessment of infants at the child health centres should always be performed when parents present worries about the infants’ amount of crying, both to rule out illness and tailor the interventions.

### Limitations

Even though the large, prospective longitudinal design of this study is an obvious strength, the results must be interpreted with caution considering some methodological limitations. Colic was assessed through one single recall question when the child was 6 months old, relying on the mother’s perception of the infant’s behaviour and her interpretation of infant crying as colic. In this study, we have no information about whether the reported colic was supported by an assessment from health professionals or not. Sleep initiation and maintenance, sleep duration, night awakenings and child temperament were also based on mothers’ perceptions. When maternal reports are the source of all measures it must be noted that they may share measurement bias, and that mother characteristics may affect their perceptions of colic, child temperament and child sleep. It is also important to note that we did not include all items of the temperament questionnaires—ICQ6 and EAS—and the use of shortened versions of the original instruments might have affected the findings and conclusions. Thirdly, multiple variables were controlled for in the analyses; however, other possible confounders, such as mothers’ mental health, maternal burden or mother-child interaction were not explored. Fourthly, a possible selection bias because of selective recruitment and attrition in the MoBa study must also be considered as a limitation [18].

## Conclusion

Our findings, based on large data, show that infant colic may appear together with other so-called regulatory problems, i.e., sleeping difficulties and problems with feeding. As excessive crying and fussing are the prominent symptoms of infant colic, colic in many cases can be viewed as a regulatory problem. It is important to be aware of and act upon symptoms of colic and other regulatory problems when we meet parents and infants in the child health centres to prevent adverse outcomes. Infants presenting with more than one regulatory problem should receive special attention because the risk of negative long-term effects increases by the number of regulatory problems. Small to moderate differences in temperamental difficulties and sleep-problems across time indicate that the challenging period of crying and fussing during the infants’ first months are moderately associated with later behavioural difficulties. However, this will depend on whether the problems persist beyond the colic period, whether the regulatory problems occur as single or multiple, and maybe most important, whether the parents get the proper support and guidance in their visits at the child health centres.

## Data Availability

The data that support the findings of this study are available from Norwegian Institute of Public Health, The Norwegian Mother, Father and Child Cohort Study (MoBa), but restrictions apply to the availability of these data, which were used under license for the current study, and so are not publicly available. Data are however available from the authors upon reasonable request and with permission of MoBa.

## Abbreviations

MoBa: The Norwegian Mother, Father and Child Cohort Study
Q: Questionnaire
ICQ: Infant Characteristics Questionnaire
EAS: The Emotionality, Activity, and Shyness questionnaire
